# Empowering community health workers in dementia care in South Texas

**DOI:** 10.1177/13872877251378463

**Published:** 2025-09-24

**Authors:** Stephanie Santiago-Mejias, Shanae L Rhodes, Fayron R Epps, Robin C Hilsabeck, Noe Garza, Sudha Seshadri, Neela K Patel

**Affiliations:** 1 Department of Neurology, Glenn Biggs Institute for Alzheimer’s and Neurodegenerative Diseases, University of Texas Health Science Center at San Antonio, San Antonio, TX, USA; 2 School of Nursing, University of Texas Health Science Center at San Antonio, San Antonio, TX, USA; 3 Institute of Neuroscience (ION), University of Texas Rio Grande Valley, School of Medicine, Harlingen, TX, USA; 4 Division of Comprehensive and Supportive Care, Glenn Biggs Institute for Alzheimer’s and Neurodegenerative Diseases, University of Texas Health Science Center at San Antonio, San Antonio TX, USA

**Keywords:** Alzheimer's disease, community health workers, dementia education, health disparities, Hispanic/Latine populations, public health

## Abstract

Alzheimer's disease and related dementias disproportionately affect minority racial and ethnic communities. Community health workers are essential in addressing these disparities. We evaluated the impact of an educational workshop focusing on improving knowledge and preparedness in dementia care. Sixty-one CHWs attended in-person sessions in Texas and completed surveys to measure confidence and plans to apply the knowledge gained. Results revealed a significant increase in confidence in providing dementia care post-training (p < 0.001). Qualitative analysis identified five primary roles of CHWs. These findings demonstrate the potential of training interventions to improve knowledge gaps and empower CHWs to address dementia in underserved communities.

## Introduction

The increasing prevalence of Alzheimer's disease and related dementias remains a public health concern, particularly in minority communities, where persistent barriers continue to limit access to healthcare.^1,2^ Community health workers (CHWs) are uniquely positioned to bridge these gaps. CHWs are trusted community members who are recognized as key contributors to dementia care teams, especially in underserved areas where resources are limited.^3–5^ Studies show how CHWs support communities and healthcare teams by navigating care, promoting health education and behavior change, and offering psychosocial support.^3,6,7^ These responsibilities correspond closely to the ten nationally recognized CHW core roles outlined by the National Council on Community Health Worker Core Consensus Standards (C3).^8^ Boughtwood and colleagues interviewed 24 bilingual/bicultural CHWs about their roles in dementia education, support, and care within culturally and linguistically diverse communities.^9^ Seven themes emerged from the interviews, including engaging with families, building trust across cultural boundaries, leveraging cultural knowledge to explain dementia, practicing flexibility in their roles, linking families to resources, and connecting communities with healthcare systems. They also addressed stigma and provided caregiver education, often extending beyond their formal job duties. De Jager and Pepper described a collaborative program in rural South Africa that trained CHWs to conduct culturally adapted dementia screenings and provide care.^10^ Through home visits, CHWs addressed barriers to care by educating families and creating action plans for social and health-related concerns.

While acknowledging the significant roles that CHWs play in the community, they often report feeling underprepared and needing additional training to confidently address dementia-related challenges, such as accurately identifying dementia symptoms (versus normal aging) and managing cognitive impairment or providing adequate support to caregivers.^11,12^ Despite these gaps, CHWs have demonstrated the ability to make meaningful contributions to dementia care when provided with appropriate training. Tailored training programs for CHWs working with Hispanic/Latine communities are particularly important, as these populations face unique sociocultural barriers, including stigma, limited health literacy, and reduced access to culturally relevant resources.^13^ To address the C3 core roles of health education, cultural mediation, and advocacy, this study builds on this foundation by evaluating the impact of a structured educational workshop, “Brain Health–Living with Dementia,” designed for CHWs who primarily engage with Hispanic/Latine communities.

## Methods

This study was determined to be exempt by the Institutional Review Board at UT Health San Antonio, as it involved anonymous, minimal risk survey research with adult participants (Protocol #20210722EX).

### Training program overview

The “Living with Dementia” training program was translated into Spanish by a certified CHW instructor and a member of the research team (BLINDED). Developed over the past decade by the senior author, the curriculum was adapted for CHWs, tailored to their community-based scope of practice, and continuously refined through an iterative, shared-decision process guided by feedback from CHWs’ annual evaluations. It has successfully trained professional and family caregivers, ensuring they are well-equipped to support individuals living with dementia. The program aims to provide participants with essential knowledge about dementia, strategies for managing the condition, and resources for supporting caregivers. Workshops are conducted using the GERIATRICS tool (Table 1) to engage participants actively and effectively: Group-Based Learning, Engaging, Relevant to Specialty, Integrated, Active Learning, Time Efficient, Reinforcing, Interactive, Centered to Person and Learner, and Supportive.^14^ This approach ensured all participants an interactive, relevant, and efficient learning experience.

**Table 1.** Components of the GERIATRICS Tool for engaging participants in training.

GERIATRICS tool component	Description
Group-Based Learning	Participants were divided into small groups (3–4 learners)
Engaging	Roundtable discussions and round-robin activities encouraged engagement
Relevant to Specialty	Emphasis on real-life stories and scenarios to ensure relevance
Integrated	Focused on integrating dementia care into the CHWs’ core roles
Active Learning	Participants worked collaboratively to create and discuss stories based on real-world scenarios
Time Efficient	Six to eight scenarios were discussed within an hour
Reinforcing	Participants were required to specify one principle they would apply in practice
Interactive	Small group discussions were followed by presentations
Centered to Person and Learner	Training principles were tailored to each participant's future specialty
Supportive	Tools and resources were shared between the facilitator and participants

The training sessions were implemented at two sites, San Antonio and the Rio Grande Valley (RGV) from March 2023 to February 2024. Sessions were conducted in person and had an average duration of approximately 4 h. The curriculum consists of five components: (a) recognizing dementia, (b) understanding the stages of dementia, (c) identifying relevant resources, (d) synthesizing key concepts through small group discussion of patient scenarios and a summary, (e) lessons learned. These components are designed to enhance participants’ knowledge and practical skills in dementia recognition, stage comprehension, and resource identification. The sessions were delivered in English in San Antonio and in English and Spanish in the RGV area, with trained facilitators leading the instruction.

### Recruitment and eligibility criteria

The training was open to certified CHWs, with recruitment facilitated through multiple channels, including the Texas Department of State Health Services newsletter, the community college CHW bulletin, and the Area Agency on Aging. Inclusion criteria required participants to be certified CHWs, aged 18 or older, fluent in English or Spanish, and willing to complete pre- and post-training assessments. Although years of CHW experience were not collected systematically, informal introductions revealed a wide range, from newly certified CHWs to individuals with roughly 15 years of practice. Individuals who chose to opt out of voluntary participation were excluded from the study. Eligible participants were invited to join the training via email and were informed of the opportunity to earn continuing education credits upon successfully completing the program.

### Study design

A pre-post training assessment was employed, where participants were asked the following question before and immediately after the training session: “How confident are you in your ability to assist families living with dementia?” (“¿Qué tan seguro está de ayudar a las familias que viven con demencia?”). Response options ranged from “1. Not at all confident” to “5. Extremely confident.” Following the training, participants were also asked, “What actions can you take as a CHW for individuals with dementia in your community?” (“¿Qué puede hacer como promotor/a de salud por las personas con demencia en su comunidad?”).

### Data analysis

Survey responses from the San Antonio cohort were all in English, while most responses from the RGV cohort were in Spanish (78%). Survey responses from both cohorts were combined for analysis as there were no *a priori* hypotheses about site differences. To assess the effect of training on confidence levels, a paired-sample t-test was conducted using SPSS software version 27 (IBM Corp., Released 2020).

An inductive content analysis was conducted to analyze the responses for the open-ended item regarding the actions CHWs can take for individuals with dementia in their community following Miles et al.^15^ Two researchers with more than five years of qualitative training and research experience carried out this analysis, one of whom was fluent in Spanish and translated responses into English when needed. Each was responsible for reviewing and independently coding the data from either the San Antonio cohort or the RGV cohort. The two researchers compared their analyses across the two cohorts and established a set of guidelines for categorizing the codes. To highlight dominant patterns, they tallied the number of excerpts assigned to each category and organized these counts in a code-frequency matrix, which also revealed co-occurring themes. The categories were presented to other team members for review and consensus validation.

## Results

**Figure 1.**
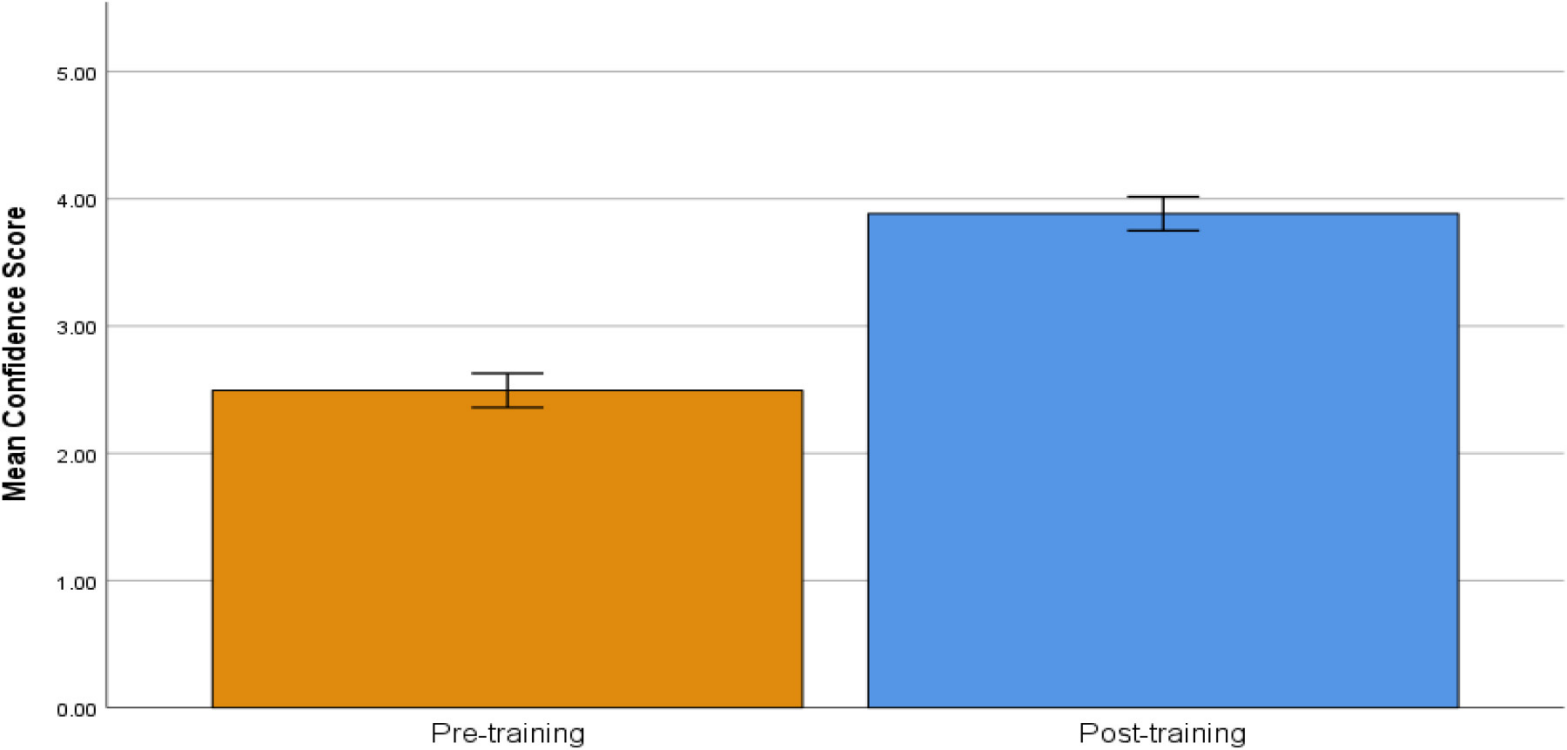
Pre-training and post-training confidence scores to the question, “How confident are you in your ability to help families living with dementia?” Response options ranged from 1 (not at all confident) to 5 (extremely confident). Error bars indicate the 95% confidence intervals (CI). Confidence scores were assessed on a scale from 1 (Not at all confident) to 5 (Extremely confident).

A total of 61 CHWs participated in the training sessions (n = 33 from San Antonio, n = 28 from RGV). For the first question, “How confident are you in your ability to help families living with dementia?”, there were seven surveys with missing data. Thus, 54 completed participant surveys were analyzed. A significant difference was found between the post-test (M = 3.89, SD = 0.74) and pre-test mean scores (M = 2.50, SD = 1.02) showing higher levels of confidence post-training with a large effect size (t(53) = −10.42, p < 0.001, Cohen's *d* = −1.42; Figure 1).

For the second question, “What can you do as a CHW for people with dementia in your community?”, responses from three participants were excluded due to either no response (n = 2) or illegible writing (n = 1), resulting in a sample size of 58. The analysis identified five distinct categories (Table 2), with the three most frequently mentioned categories being: providing education/information, sharing resources, and providing support to families and communities.

**Table 2.** Categories and frequencies for what CHWs can do for people with dementia in their community.

Category	Frequency n (%)	Quotes
Provide education/information	37 (64%)	“Provide education to families in need of knowledge and understanding of this disease. Keep families informed of updates, information, and resources available to them.” “Compartir y facilitar información para poder ayudar a las personas que presentan signos o que ya tienen demencia.” [*Share and provide information to help individuals showing signs of or already diagnosed with dementia.*]
Share resources	28 (48%)	“Provide them with resources. Connect them to support groups.” “Guia[r] a las personas hacia centros y recursos de ayuda con profesionales de la salud especialistas en demencia y Alzheimer.” [*Guide individuals to centers and resources that offer support and healthcare professionals specializing in dementia and Alzheimer's.*]
Provide support to families and communities	16 (28%)	“[I can] support families with care coordination, clinical research/trials, appointment … and senior centers.” “Tenerles paciencia, brindarle apoyo para cubrir sus necesidades, revalorizarlos.” [*Have patience, provide support to meet their needs, and help restore their sense of value.*]
Gain skills and knowledge	13 (22%)	“Take trainings to learn more on how to better cope/care for a loved with dementia.” “Prepararme para conocer más de esta condición y conocer los recursos de mi comunidad que atiendan este padecimiento.” [*Prepare myself to learn more about this condition and become familiar with the community resources available to address it.*]
Advocate	9 (16%)	“Be a resource and advocate.”

Most CHWs (64%; n = 37) indicated that they can provide education and information to individuals with dementia in their community. Participants felt equipped to disseminate information to help the community understand dementia, including its early symptoms and signs. Almost half of participants (48%, n = 28) reported that they can share resources (e.g., dementia-specific support groups), and approximately one-third (28%; n = 16) expressed that they can support families and communities by assisting with care coordination, identifying clinical research trials, scheduling appointments, locating senior centers, and managing individuals living with dementia. More than 20% (n = 13) considered gaining knowledge and skills about dementia as part of their responsibility, which includes continuing education, participating in dementia care training, and staying informed about the latest developments in Alzheimer's disease. A smaller proportion of participants (16%, n = 9) identified advocacy for individuals affected by dementia as another key aspect of their role.

## Discussion

This study evaluated the impact of a training workshop designed to provide CHWs with education for addressing dementia-related health needs in Hispanic/Latine communities. Results revealed an increase in CHWs’ self-reported confidence levels, which improved from “slightly” to “moderately confident” before the training to “moderately” to “very confident” afterward. Findings demonstrate the potential of training interventions to improve gaps in knowledge and empower CHWs to provide education in dementia and support to historically underserved communities.

Content analysis of participant responses after the training identified the most common roles that CHWs are likely to assume in their communities, including providing education, sharing resources, supporting families and the community, gaining knowledge, and advocacy. Most participants emphasized their ability to provide education and information about dementia, particularly in raising awareness of early symptoms and fostering a better understanding of the disease. This echoes previous research on the contributions of CHWs in delivering dementia-related education and support.^6,16^ Participants also reported supporting families and communities by assisting with care coordination, locating clinical trials, scheduling appointments, and addressing other practical needs. This diverse range of responsibilities makes CHWs essential in reducing the burden on caregivers, a finding consistent with the growing recognition of CHWs as integral members of healthcare teams.^16^ Over 20% emphasized the importance of gaining and maintaining knowledge about dementia, reflecting the value of ongoing education for strengthening their readiness to make meaningful contributions within their communities. Other roles identified by participants, such as advocacy, are essential in addressing systemic barriers to care and ensuring that underserved populations receive equitable access to resources. The education, care-coordination, outreach, and advocacy activities reported by our participants map directly onto several of the CHW core roles established by the National C3 Council.^8^ Results of our study suggest that tailored training programs not only improve CHWs’ confidence but also prepare them to address the needs of communities affected by dementia.

Findings build upon prior evidence demonstrating the importance of tailored training programs in preparing CHWs to facilitate earlier intervention and support in their communities. For example, Reinschmidt and colleagues developed and evaluated a CHW-focused dementia care curriculum in Oklahoma, which used a train-the-trainer and virtual approach to train 77 CHWs.^17^ The program improved participants’ dementia knowledge and emphasized the importance of peer-led instruction and culturally tailored resources. Similarly, de Jager and Pepper emphasized the success of collaborative efforts in resource-constrained settings by implementing culturally appropriate dementia screening and care in rural South Africa.^10^ Our findings extend this understanding to Hispanic/Latine communities in the United States, which present distinct cultural and contextual differences. These communities face unique barriers to dementia care, including limited health insurance, transportation challenges, and language barriers.^18^ Additionally, cultural misconceptions exacerbate these challenges, as many Hispanic/Latines perceive dementia as a normal part of aging and believe they will not live long enough to develop Alzheimer's disease.^13^ These beliefs often lead to delays in diagnosis and intervention, making CHWs critical assets in addressing these challenges.

This study had a short-term follow-up period, which limits the assessment of the long-term retention of knowledge CHWs gained through the training workshop. Additionally, we did not collect CHWs years of experience, limiting our ability to assess its impact on training outcomes. Future research should include long-term assessments and evaluations to determine if CHWs continue to feel prepared for their roles after an extended period of time. CHWs have significant potential as advocates in the care of individuals with dementia and expanding structured training programs for continuing education credits to other topics, such as conducting cognitive, emotional, and functional assessments, can further enhance their impact on their communities. Future studies can expand on these findings by exploring the longitudinal effects of dementia-focused educational training workshops for CHWs on community health outcomes.

## References

[1] Hudomiet P Hurd MD Rohwedder S . Trends in inequalities in the prevalence of dementia in the United States. Proc Natl Acad Sci U S A 2022; 119: e2212205119. 10.1073/pnas.2212205119 PMC9674270 36343247

[2] Manly JJ Jones RN Langa KM , et al. Estimating the prevalence of dementia and mild cognitive impairment in the US: the 2016 health and retirement study harmonized cognitive assessment protocol project. JAMA Neurol 2022; 79: 1242. 36279130 10.1001/jamaneurol.2022.3543 PMC9593315

[3] Alam RB Ashrafi SA Pionke JJ , et al. Role of community health workers in addressing dementia: a scoping review and global perspective. J Appl Gerontol 2021; 40: 1881–1892. 33736506 10.1177/07334648211001190

[4] Patel N Rodriguez J Davila A , et al. Clinicians and promotorés teaming to provide in-home primary care to frail older adults. Ann Fam Med 2023; 21: 377. 37487729 10.1370/afm.2998 PMC10365879

[5] Petry SE Lara L Boucher NA . Older caregivers: who they are and how to support them. J Aging Soc Policy 2024; 36: 589–602. 35290168 10.1080/08959420.2022.2051683

[6] Alinaitwe R Musisi S Mukunya D , et al. Feasibility of screening for cognitive impairment among older persons and referral by community health workers in Wakiso district, Uganda. BMC Psychiatry 2023; 23: 533. 37488506 10.1186/s12888-023-05015-0 PMC10367281

[7] World Health Organization . *What do we know about community health workers? A systematic review of existing reviews*, https://www.who.int/publications-detail/what-do-we-know-about-community-health-workers-a-systematic-review-of-existing-reviews (2020).

[8] Rosenthal E Menking P St. John J , et al. https://www.c3council.org/ .

[9] Boughtwood D Shanley C Adams J , et al. The role of the bilingual/bicultural worker in dementia education, support and care. Dementia 2013; 12: 7–21. 24336659 10.1177/1471301211416173

[10] De Jager CA Pepper K . Building capacity for dementia screening and intervention in low income, rural communities: a collaborative initiative. Int J Afr Nurs Sci 2017; 7: 43–49.

[11] Spadoni N Baron A Zavala E , et al. Community health worker training on older adults: a qualitative needs assessment. J Am Geriatr Soc 2024; 72: 2825–2833. 39016122 10.1111/jgs.19077 PMC11368648

[12] Wang Y Xiao LD Luo Y , et al. Community health professionals’ dementia knowledge, attitudes and care approach: a cross-sectional survey in Changsha, China. BMC Geriatr 2018; 18: 122. 29801476 10.1186/s12877-018-0821-4 PMC5970511

[13] Quiroz YT Solis M Aranda MP , et al. Addressing the disparities in dementia risk, early detection and care in Latino populations: highlights from the second Latinos & Alzheimer’s symposium. Alzheimers Dement 2022; 18: 1677–1686. 35199931 10.1002/alz.12589 PMC9399296

[14] Patel N Chidinma A Davis V . GERIATRICS-A 4th year course to help medical students apply geriatric care in any specialty. J Am Geriatr Soc 2020; 70: S208–S209.

[15] Miles MB Huberman AM Saldaña J . Qualitative data analysis: a methods sourcebook. 3rd ed. Thousand Oaks, CA: Sage Publications, Inc, 2014.

[16] Hunt JFV Caire TJL Schroeder M , et al. The use of healthcare and community-based services by people living with dementia and their caregivers during the COVID-19 pandemic. WMJ 2022; 121: 226–230. 36301650 PMC9799242

[17] Reinschmidt KM Philip TJ Alhay ZA , et al. Training community health workers to address disparities in dementia care: a case study from Oklahoma with national implications. J Ambulatory Care Manage 2023; 46: 272–283. 36939639 10.1097/JAC.0000000000000470

[18] Aranda MP Marquez DX Gallagher-Thompson D , et al. A call to address structural barriers to Hispanic/Latino representation in clinical trials on Alzheimer’s disease and related dementias: a micro-meso-macro perspective. Alzheimers Dement (N Y) 2023; 9: e12389. 10.1002/trc2.12389 PMC10242183 37287471

